# Identification of Potential Repurposable Drugs in Alzheimer’s Disease Exploiting a Bioinformatics Analysis

**DOI:** 10.3390/jpm12101731

**Published:** 2022-10-18

**Authors:** Giulia Fiscon, Pasquale Sibilio, Alessio Funari, Federica Conte, Paola Paci

**Affiliations:** 1 Department of Computer, Control and Management Engineering, Sapienza University of Rome, 00185 Rome, Italy; 2 Institute for Systems Analysis and Computer Science “Antonio Ruberti”, National Research Council, 00185 Rome, Italy; 3 Department of Translational and Precision Medicine, Sapienza University of Rome, 00185 Rome, Italy

**Keywords:** network theory, drug repurposing, dementia

## Abstract

Alzheimer’s disease (AD) is a neurologic disorder causing brain atrophy and the death of brain cells. It is a progressive condition marked by cognitive and behavioral impairment that significantly interferes with daily activities. AD symptoms develop gradually over many years and eventually become more severe, and no cure has been found yet to arrest this process. The present study is directed towards suggesting putative novel solutions and paradigms for fighting AD pathogenesis by exploiting new insights from network medicine and drug repurposing strategies. To identify new drug–AD associations, we exploited SAveRUNNER, a recently developed network-based algorithm for drug repurposing, which quantifies the vicinity of disease-associated genes to drug targets in the human interactome. We complemented the analysis with an in silico validation of the candidate compounds through a gene set enrichment analysis, aiming to determine if the modulation of the gene expression induced by the predicted drugs could be counteracted by the modulation elicited by the disease. We identified some interesting compounds belonging to the beta-blocker family, originally approved for treating hypertension, such as betaxolol, bisoprolol, and metoprolol, whose connection with a lower risk to develop Alzheimer’s disease has already been observed. Moreover, our algorithm predicted multi-kinase inhibitors such as regorafenib, whose beneficial effects were recently investigated for neuroinflammation and AD pathology, and mTOR inhibitors such as sirolimus, whose modulation has been associated with AD.

## 1. Introduction

Alzheimer’s disease (AD) is a progressive neurodegenerative disease, characterized by a pathological accumulation of β-amyloid-containing plaques and tau-containing neurofibrillary tangles. It is the most common form of dementia that affects memory, thinking, behavior, and other cognitive abilities, and is serious enough to interfere with daily life [1]. The greatest known risk factor is increasing age, and the majority of people with AD are 65 years and older, where dementia symptoms gradually worsen over a number of years [2]. In its early stages, memory loss is mild, but with late-stage, individuals lose the ability to carry on a conversation and respond to their environment. AD is the sixth-leading cause of death in the United States, and it still has no cure, meaning that once this disease damages the brain, it is very difficult to overcome the loss of memory and mental functions [2]. Early diagnoses and interventions are urgently needed to improve quality of life and to either slow or halt the course of the disease.

In this framework, a promising strategy relies on drug repurposing, aiming to identify novel uses for drugs approved by the US Food and Drug Administration (FDA) outside the scope of their original medical indications [3,4,5]. The process of redeveloping a compound for use in a different disease has been established as a time-saving and cost-efficient method with respect to de novo drug discovery, and has resulted in higher success rates, which can therefore drastically reduce the risk of drug development and offer new solutions for neurodegenerative diseases with no cure such as AD. The growing interest in this field has led to the development of novel computational methodologies in recent years, such as those exploiting the new emerging paradigm of network medicine [6,7,8].

Recently, we developed a network-based algorithm for drug repurposing, SAveRUNNER [9,10], which has been successfully applied to identify candidate repurposable drugs for several diseases, including neurodevelopmental ones such as amyotrophic lateral sclerosis and multiple sclerosis [11,12]. SAveRUNNER exploits the network medicine principles and quantifies the proximity of disease-associated genes to drug targets in the human interactome via the computation of a novel network-based similarity measure that awards those drug targets and disease genes that fall in the same neighborhood [9,10].

In the present work, we exploited SAveRUNNER to unveil candidate repurposable solutions for AD and its related diseases. In particular, we studied 13 AD-related diseases, including mild cognitive impairment (MCI), which is the stage between the expected cognitive decline of normal aging and the more serious decline of dementia; other neurological diseases (i.e., Parkinson’s disease, Huntington disease, supranuclear palsy progressive, and Lewy body disease); and other diseases that can be symptoms, risk factors, or consequences of AD (i.e., language disorders, memory disorders, visual pattern recognition, executive dysfunction, choice behavior, cardiomyopathy, and amyloid neuropathies).

**Figure 1:**
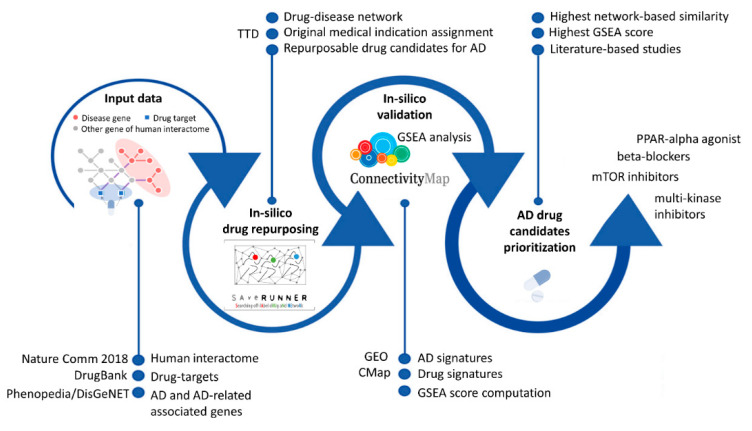
Workflow of the study. The input data are: (i) the human interactome download from [6], (ii) a list of drug targets acquired from DrugBank [19]; (iii) a list of disease-associated genes for Alzheimer’s disease (AD) and other diseases related to AD acquired from the Phenopedia [20] and DisGeNET [21] data sources. First, an in silico drug repurposing analysis was performed by using the SAveRUNNER algorithm to obtain candidate drugs for the understudied diseases. SAveRUNNER releases the drug-disease network, where the nodes are drugs and diseases, and a link occurs between them if the drug–disease association has been found to be statically significant and the weight of their association is the network-based similarity measure. Then, the original medical indications are retrieved from Therapeutic Target Database (TTD) and are assigned to each predicted drug. Looking at the drugs predicted by SAveRUNNER for AD, an in silico validation based on the gene set enrichment analysis (GSEA) has been performed by assigning a score that reflects the possible counteraction of the predicted drugs (drug signatures acquired from Connectivity Map—CMap [22,23]) on the AD pathophenotype (disease signatures acquired from the Gene Expression Omnibus—GEO [24]).

Finally, we complemented the analysis with an in silico validation of the candidate compounds predicted by SAveRUNNER through the gene set enrichment analysis (GSEA), aiming to determine if the modulation of the gene expression induced by the predicted drugs could be counteracted by the modulation elicited by the disease (Figure 1). Our analysis highlighted some interesting compounds worthy of further investigation, such as those belonging to the family of beta-blockers, originally used for hypertension treatment and already linked to a lower risk of developing AD [13]; sirolimus, also known as rapamycin, an immunosuppressant drug used in transplantation medicine to prevent organ rejection, whose modulation could have potential application to delay memory loss and age-related neurodegenerative disease, such as AD [14,15]; clopidogrel, belonging to the anti-platelet inhibitor family, whose therapy has been associated with a lower risk of developing AD [16] and whose anti-inflammatory effect has been recently studied in rat model of AD [17]; or regorafenib, a multi-kinase inhibitor, whose beneficial effects were recently investigated on neuroinflammation, AD pathology, and dendritic spine formation in vitro and in vivo [18].

## 2. Materials and Methods

### 2.1. Data Retrieval

#### 2.1.1. Human Interactome

The human protein–protein interactome was downloaded from [6], where the authors assembled their in-house systematic human protein–protein interactome with 15 commonly used databases supported by several types of experimental evidence (e.g., binary PPIs from three-dimensional protein structures; literature-curated PPIs identified by affinity purification followed by mass spectrometry; Y2H, and/or literature-derived low-throughput experiments such as BioGRID [25], HPRD [25], MINT [26], IntAct [25], and InnateDB [25]; signalling networks from literature-derived low-throughput experiments; and kinase-substrate interactions from literature-derived low-throughput and high-throughput experiments). This version of the interactome comprises 217,160 protein–protein interactions connecting 15,970 unique proteins.

#### 2.1.2. Drug–Target Interactions

Drug–target interactions were acquired from DrugBank [19], which contains 13,563 drug entries, including 2627 approved small molecule drugs, 1373 approved biologics, 131 nutraceuticals, and over 6370 experimental drugs (version 5.1.6 released on 22 April 2020). The targets’ Uniprot IDs provided by DrugBank were mapped to Entrez gene IDs by using the BioMart–Ensembl tool (https://www.ensembl.org/ (accessed on 18 October 2021)), yielding a total of 2165 genes interacting with 1873 drugs.

#### 2.1.3. Drug Medical Indications

The original approved medical indications for the drugs were acquired from the Therapeutic Target Database (TTD) [27] (last version released on 1 June 2020), which includes information about 5059 drugs associated with 1136 disease classes.

#### 2.1.4. Disease Gene Associations

Disease-associated genes were downloaded from Phenopedia (last release April 2020) [20] and DisGeNET (last release October 2021) [21], two of the more popular publicly available collections of disease genes obtained by integrating and homogeneously annotating data from expert-curated repositories and GWAS catalogues. In particular, Phenopedia provides a curated collection of gene–disease associations retrieved from PubMed and summarized in the Human Genome Epidemiology (HuGE) encyclopedia [28]. DisGeNET provided a curated collection of gene–disease associations integrated from UniProt, PsyGeNET, Orphanet, the The Cancer Genome Interpreter (CGI), Comparative Toxicogenomics Database (CTD), ClinGen, and the Genomics England Panel App [21].

In particular, we selected a panel of 14 diseases of interest with their associated genes (Table 1).

### 2.2. SAveRUNNER Algorithm

SAveRUNNER (Searching off-lAbel dRUg aNd NEtwoRk) [9,10] is a novel network-based algorithm for drug repurposing that we recently developed with the aim of screening efficiently novel potential indications for currently marketed drugs against diseases of interest, and for optimizing the efficacy of putative validation experiments. Taking the human interactome network as the input, the list of disease–gene associations, and the list of drug–target interactions, SAveRUNNER predicts drug–disease associations by quantifying the interplay between the drug targets and disease-associated proteins in the human interactome via a novel network-based similarity measure (denoted adjusted similarity), defined as follows:

(1) $$AS\left(p\right)=\frac{1}{1+e^{-c\left[\frac{\left(1+QC\right)\left(m-p\right)}{m}-d\right]}}$$

where *p* is the network proximity measure defined in [6]:

(2) $$p\left(T,S\right)=\frac{1}{T}\underset{t\epsilon T}{\sum }\underset{s\epsilon S}{\min}\;d(t,s)$$

*p*(*T*, *S*) represents the average shortest path length between drug targets t in the drug module T and the nearest disease genes s in the disease module S; QC is a quality cluster score that rewards associations between drugs and diseases located in the same network cluster; m is max(p); and c  and d are the steepness and the midpoint of AS(p), respectively. A comprehensive description of the SAveRUNNER methodology can be found in [9,10].

### 2.3. In Silico Validation: Gene Set Enrichment Analysis

To test whether the drugs predicted as being repurposable drugs for AD could counteract the gene expression perturbations caused by AD, we performed a Gene Set Enrichment Analysis (GSEA). This analysis may represent a sort of in silico validation of the algorithm predictions and takes the following as inputs: (i) differentially expressed genes of AD patients and control samples to use as “disease signature”, and (ii) differentially expressed genes of drug-treated human cell lines to use as “drug signature”.

#### 2.3.1. Disease Signature

We first collected three gene expression datasets of AD patients and controls samples available through the GEO public repository [24]. In particular:

(i) Expression profiling by an array of human frozen hippocampal tissue blocks containing both gray and white matter from a total of 30 subjects, including 22 patients with AD and 8 control samples (GSE28146 [29]).
(ii) Expression profiling by an array of entorhinal cortex neurons from 19 AD patients and 14 non-demented controls (GSE4757 [30]).
(iii) Expression profiling by array related to AD and control samples originating from the EU funded AddNeuroMed Cohort [31] and available from the GEO repository via the following accession numbers: GSE63060—batch 1 and GSE63061—batch 2 [32]. In particular, batch 1 (GSE63060) has a total of 249 samples, including 145 AD and 104 control samples, whereas batch 2 (GSE63061) has a total of 273 samples, including 139 AD and 134 control samples. The probe-sets were mapped to official gene symbols using the relative platform (GPL6947-13512 for GSE63060 and GPL10558-50081 for GSE63061). Multiple probe measurements of a given gene were collapsed into a single gene measurement by considering the mean. By matching genes based on gene symbols, we created a single merged dataset with both batches. We ran Combat function from R/Bioconductor package SVA to correct for batch-specific effects. Finally, we obtained a data matrix of 19,460 gene symbols (rows) and 522 samples (columns) including 284 AD and 238 control samples.

The first two datasets, GSE28146 and GSE4757, were analyzed through the GEO analyzer tool available from the GEO repository. The third built-up dataset (i.e., GSE63060 + GSE63061) was processed by applying a logarithmic (log2) transformation of the expression values, and by conducting a preprocessing analysis via the computation of the inter quartile range (IQR) for each gene (i.e., equal to the difference between the 75th and 25th percentiles of the data distribution, measuring data variability around the median). Those genes with an IQR value smaller than the 10th percentile of the IQR distribution (corresponding to those genes less scattered around the median) were filtered out. Then, we performed the unpaired Student’s *t* test and we adjusted the obtained *p*-values for multiple hypothesis testing by using the Benjamini–Hochberg procedure.

In order to select statistically significant differentially expressed genes, we set a threshold of 0.01 on the adjusted *p*-values for the dataset with the largest number of samples (i.e., GSE63060 + GSE63061). For the other two datasets (i.e., GSE28146 and GSE4757), we obtained adjusted *p*-values that had always been greater than the standard significant level. Thus, only for these datasets, we decided to discard the adjustment of the *p*-values and to compensate for this shortcoming by using a more severe threshold of 0.01 on the original *p*-values.

We used the three lists of differentially expressed genes as the AD signatures.

#### 2.3.2. Drug Signature

We exploited the Connectivity Map (CMap) database that catalogs high-throughput transcriptional responses of human cells to chemical and genetic perturbation obtained by using an L1000 assay, thus collecting a variety of drug-treated human cell lines [22,23]. A total of 27,927 perturbagens have been profiled in a core set of nine cell lines to produce 476,251 expression signatures.

In particular, among the available cell lines from the CMap database, we selected human iPS-derived neural progenitor cell line (NPC) and neuron cells terminally differentiated in-plate from NPC cell lines (NEU), and we used the differentially expressed genes of those drug-treated human cell lines as the drug signatures.

#### 2.3.3. GSEA Score Computation

For each drug that was in both the CMap database and predicted by SAveRUNNER to be repurposable for AD, we calculated a score as an indication of its possible counteraction to the gene expression perturbations caused by AD pathophenotype, by exploiting CMap query tool [22]. In particular, the disease signature and the drug signature were ordered by increasing the fold change, and then the CMap query tool computed an enrichment score (ES) that measures whether the effect of the drug could counteract the effect of the disease (ES < 0) or not (ES > 0) [23,33]. The idea underlying this procedure is the following: one ordered list of genes characterizing the disease signature is compared with one ordered list of genes characterizing the drug signature for determining if the highest down-regulated (up-regulated) gene in the disease signature is near the top (bottom) of the drug signature. This would mean that the disease and the drug have complementary expression profiles (ES < 0), and the drug could be a possible treatment option for the disease of interest. More details on the computation of this score are given in [23,33,34].

A selected candidate repurposable drug was considered to have a potential treatment effect against AD if the drug signature was negatively correlated with the tested AD signature. We stated that a disease and a drug were negatively correlated if the corresponding ES was negative, and we assigned a score equal to 1 to that drug for that disease signature. Finally, for each drug, the so-called GSEA score was equal to the number of AD datasets satisfying this criterion. This final GSEA score ranged from 1 to *n*, where *n* is the total number of AD signatures tested. By adopting three AD datasets in this study (i.e., three AD signatures), for each drug, the maximum GSEA score will be equal to 3, meaning that the drug showed an ES < 0 with all of the three analyzed AD signatures, and thus its potential effect for treating AD was more corroborated with respect to a GSEA score equal to 2 or 1. Finally, a GSEA score equal to “not available” was assigned to all those drugs that were not included in the CMap database or not available for a specific cell line of interest.

## 3. Results and Discussion

### 3.1. Drug–Disease Network

The SAveRUNNER algorithm [9,10] has been applied to find repurposable solutions for AD and 13 other AD-related diseases. SAveRUNNER requires a list of drug targets and a list of disease genes to evaluate the extent to which a given drug can be eventually repositioned to treat a disease as an input. Here, the disease-associated genes were downloaded from Phenopedia [20] and DisGeNET [21], whereas drug–target associations were obtained from DrugBank [19]. In particular, we assembled target information about a total of 1873 FDA-approved drugs.

The rationale behind SAveRUNNER lies in the hypothesis that, for a drug to be effective against a specific disease, its associated targets (drug module) and the disease-specific associated genes (disease module) should be nearby in the human interactome [6]. To quantify the vicinity between drug and disease modules, SAveRUNNER implements a new network similarity measure and assesses its statistical significance by applying a degree-preserving randomization procedure [9]. Its novelty relies on the implementation of a procedure to prioritize the predicted off-label drugs for a given disease, which is based on a clustering analysis to reward associations between drugs and diseases belonging to the same cluster. This procedure is based on the assumption that if a drug and a disease are grouped together, most likely that drug can be effectively be repurposed for that disease.

As the output, SAveRUNNER releases a weighted bipartite drug–disease network, where nodes are drugs and diseases, and a link between a drug and a disease occurs if the corresponding drug targets and disease genes are closer in the interactome than expected by chance. The weight of their interaction corresponds to the network-based similarity measure.

**Figure 2:**
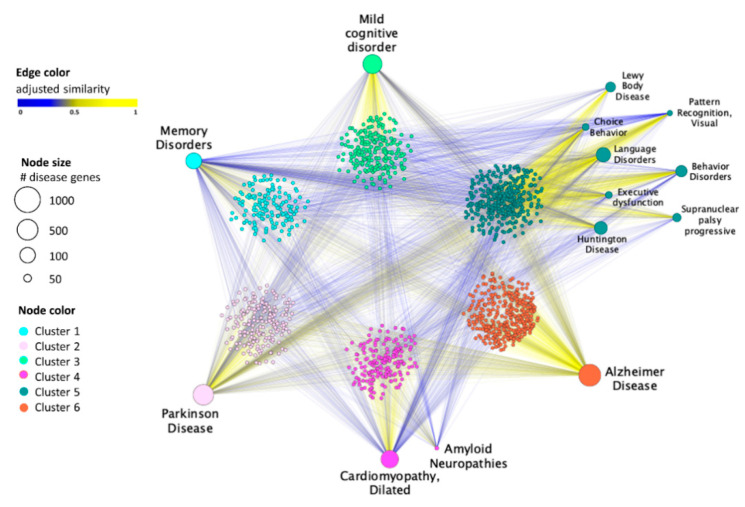
Drug–disease network. This graph shows the high-confidence predicted drug–disease associations connecting 14 diseases (bigger labelled circles) with the 1468 FDA-approved drugs (smaller circles). The node sizes are scaled with the number of genes associated to each analyzed disease. The edge color denotes the adjusted similarity between the drug targets and disease genes mapped on the human interactome, increasing from blue (less similar) to yellow (more similar). The node color refers to one of the six clusters identified by SAveRUNNER according to the label reported in the legend. For the clusters’ identification on the drug–disease network, SAveRUNNER exploits a cluster detection algorithm based on greedy optimization of the network modularity [35].

In this study, the final drug–disease network was composed of a total of 1482 nodes (i.e., 14 diseases associated with 1468 drugs) and 5333 edges (Figure 2). Each node representing a disease was scaled according to the number of its associated genes, while each edge of this network was colored according to the corresponding similarity value of a given drug–disease pair: shades of yellow denote drug-associated targets more proximal (high similarity) to the disease-associated genes in the human interactome, whereas shades of blue denote drug targets more distal (low similarity) to the disease genes.

SAveRUNNER makes use of a modularity-based greedy optimization clustering algorithm that, exploiting the geometry of the drug–disease network, highlights six different clusters (Figure 2 and Supplementary Table S1).

In particular, we observed that AD belongs to one separate cluster, while the other risk factors, such as amyloid neuropathies and cardiomyopathy, as well as other symptoms or AD consequences, including behavior disorders, choice behavior, executive dysfunction, Huntington’s disease, Lewy Body disease, language disorders, pattern recognition, and supranuclear palsy progressive, were grouped in other separated clusters.

**Figure A1:**
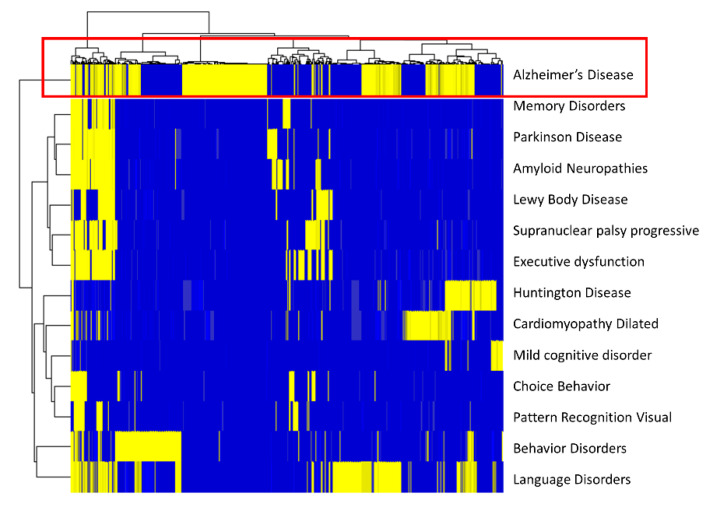
Dendrogram and heatmap of the drug-disease network. The network adjusted similarity values are clustered according to rows (diseases) and columns (drugs) by using Ward’s minimum variance method as linkage hierarchical clustering algorithm and by using the Euclidean distance as distance metric. Heatmap color key denotes the adjusted and normalized network similarity between drug targets and disease genes in the human interactome, increasing from blue (less similar) to red (more similar).

The drug–disease network was then rendered as an heatmap reporting the 14 diseases in the rows, and the 1468 drugs in the columns, where each cell was colored according to the corresponding similarity value of a given drug–disease pair (Figure A1). By computing a hierarchical biclustering on the drug–disease similarity matrix, we pointed out two main disease clusters: one including Alzheimer’s disease, and the other one including other neurological diseases with their symptoms, consequences, and risk factors (Figure A1).

**Figure A2:**
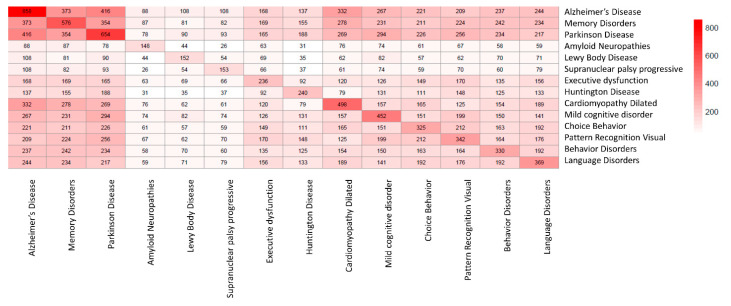
Number of repurposable drugs. Heatmap showing the number of drugs predicted to be repurposable for each disease (on the diagonal) and the number of drugs shared among each pair of diseases. Heatmap color scales with the number of drugs, increasing from white to red.

Both in the hierarchical clustering and the greedy cluster implemented by SAveRUNNER, Alzheimer’s disease belonged to a separate cluster with respect to the other diseases, suggesting that more treatments are specifically predicted for AD than shared with other diseases. In particular, among the total 858 repurposable drugs that were identified by SAveRUNNER as being associated with AD (Supplementary Table S2 and Figure A2), 133 drugs were specifically predicted only for AD treatment (Supplementary Table S2).

**Figure 3:**
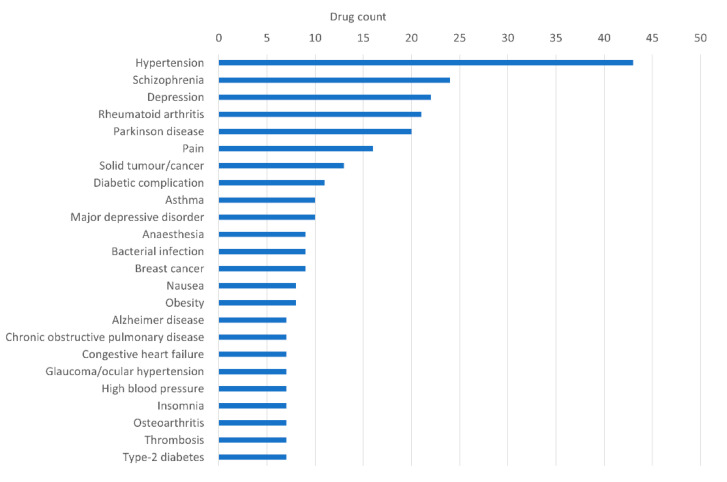
Distribution of the original medical indications of drugs predicted to be repurposable for AD. Bar plot of the top 30 original medical indications with at least five associated drugs, retrieved from TTD [27].

By analyzing the distribution of all the known medical indications associated to the drugs predicted for AD, hypertension- and schizophrenia-associated drugs appeared as highly frequent drugs, followed by depression, rheumatoid arthritis, and Parkinson’s disease-associated ones (Figure 3 and Supplementary Table S2). Among the drugs predicted by SAveRUNNER originally used for hypertension treatment with a high adjusted similarity value (i.e., >0.8), we highlighted those belonging to the family of beta adrenergic blocking agents (i.e., acebutolol, atenolol, betaxolol, bisoprolol, metoprolol, and nebivolol), already linked to a lower risk of developing AD [13]. As proof of the validity of SAveRUNNER, we found also seven compounds already used for treating patients affected by Alzheimer’s disease, which are donepezil, ergoloid mesylate, huperzine a, rivastigmine, galantamine, memantine, and tacrine (Figure 3 and Supplementary Table S2). On the other hand, for aducanumab, a monoclonal antibody approved for the treatment of mild-stage AD [36] by the FDA and not by the European Medicines Agency (EMA), no sufficient data were present to assign a statistical significance to its association with AD.

### 3.2. In Silico Validation: GSEA Analysis

To validate the repurposed drugs predicted by SAveRUNNER for fighting AD in silico, we performed a gene set enrichment analysis (GSEA) that allowed us to evaluate the treatment effects on those genes associated with the AD pathophenotype (cf. Materials and Methods). In order to complete this task, two main inputs are required: (i) differentially expressed genes of AD retrieved from the GEO database to use as disease signature; (ii) differentially expressed genes of drug-treated human cell lines from the Connectivity Map (CMap) [23] database to use as drug signature. Here, for each drug, the following three disease signatures (i.e., three different datasets associated to AD) had been tested: (1) differentially expressed genes from GSE28146 [29], with 140 up-regulated genes and 248 down-regulated genes; (2) differentially expressed genes from GSE4757 [30], with 34 up-regulated genes and 88 down-regulated genes; and (3) differentially expressed genes from GSE63060 + GSE63061 [32], with 1846 up-regulated genes and 1512 down-regulated genes. For the drug signatures, we selected the neural drug-treated cell lines available from CMap (i.e., NPU and NSE).

For each drug predicted by SAveRUNNER and included in the CMap database, we calculated a GSEA score as an indication of its possible counteraction to the gene expression perturbations caused by the AD pathophenotype. In particular, for each AD dataset, we selected drugs whose signatures were negatively correlated with the AD signature according to the CMap query tool [22] as being able to have a potential treatment effect against genes associated to AD (cf. Materials and Methods). The GSEA score, ranging from 1 to 3, corresponded to the number of AD datasets satisfying this criterion for a specific drug.

The validation analysis highlighted a total of 155 out of the 858 candidate drugs with an available GSEA score, including six drugs with a GSEA equal to 3, 44 with a GSEA score equal to 2, and 105 with a GSEA score equal to 1 (Supplementary Table S2). In the following, we detailed and discuss some of the candidate drugs summarized in Table 2.

#### 3.2.1. GSEA Score 3

The drug specifically predicted for AD with a high adjusted similarity and corroborated by a GSEA score equal to 3 was tamoxifen, a selective estrogen receptor modulator that inhibits growth and promotes apoptosis in estrogen receptor positive tumors, that was thus originally approved for treating estrogen receptor positive breast cancer. Interestingly, a recent study showed the association between hormone-modulating breast cancer therapy and the decreasing incidence of dementia, showing that among patients with breast cancer, tamoxifen and steroidal aromatase inhibitors were associated with a decrease in the number of patients who received a diagnosis of dementia, specifically Alzheimer’s disease [37].

Among the drugs showing a GSEA equal to 3, we found also regorafenib, a kinase inhibitor used to treat patients with metastatic colorectal cancer, and dexamethasone, a glucocorticoid used to treat various inflammatory conditions including bronchial asthma, endocrine, and rheumatic disorders. Recently, a research study investigated the beneficial effects of regorafenib on neuroinflammation and AD pathology in vitro and vivo, showing that regorafenib-injection displayed significant suppression of Aβ plaque levels and tau phosphorylation in a mouse model of AD [18]. It has been also found that the short-term use of dexamethasone could inhibit AD-related neuroinflammation. In particular, the authors of [38] showed that combined treatment with acyclovir in an AD mouse model could prevent β-amyloid (Aβ) oligomer-induced spatial cognitive impairments. This treatment could also avoid neuroinflammation due to Aβ oligomer-induced over-activation of the microglia and astrocytes, and an overexpression of the pro-inflammatory cytokines [38].

#### 3.2.2. GSEA Score 2

Other drugs specifically predicted for AD with a high adjusted similarity and showing a GSEA score equal to 2 were clopidogrel and gemfibrozil. Clopidogrel is a prodrug of a platelet inhibitor used to reduce the risk of myocardial infarction and stroke (Table 2). The anti-platelet treatment has recently been associated with a lower risk of AD [16], and has been studied to treat neuroinflammation in a rat model of AD [17]. Gemfibrozil is a PPARα agonist and FDA-approved drug for hyperlipidemia treatment, used to reduce serum triglyceride levels by influencing lipid metabolism through the activation of PPARα. It has been shown that gemfibrozil leads to a reduction in the load of cerebral amyloid-beta plaques in a 5XFAD mice model of AD [39]. This neuropathological event is one of the most important characterizing AD, and the authors of [39] showed that this drug could reduce neuroinflammation by reducing glial activation and could improve memory in 5XFAD mice. Furthermore, another research study showed that gemfibrozil is capable of reducing amyloid pathology and reversing memory deficits and anxiety symptoms in another mouse model of AD [40]. Its administration in the mouse model determines a reduction in soluble Aβ and insoluble Aβ in the hippocampus and cortex tissues. The motivation would lie in PPARA’s ability to regulate autophagy in the clearance of Aβ, though the recruitment of microglia and astrocytes [40].

Another interesting compound showing a high adjusted similarity and a GSEA score equal to 2 was sirolimus, also known as rapamycin, an mTOR inhibitor immunosuppressant drug, used in transplantation medicine to prevent organ rejection, to treat lymphangioleiomyomatosis and perivascular epithelioid cell tumors (Table 2). Its modulation could have potential application to delay age-related neurogenerative disease, including AD [14], as well as to prevent or restore memory deficit, as observed in a mouse model of AD [15]. This finding appears in accordance with the fact that this drug was predicted by SAveRUNNER both for Alzheimer’s disease and memory disorders.

Another mTOR inhibitor specifically predicted for AD and with the highest adjusted similarity was everolimus, a derivative of sirolimus, which was developed to improve its pharmacokinetic characteristics. The administration of everolimus in an AD mouse model has been shown to have positive effects for AD-like phenotypes [41]. Short-term intrathecal infusion leads to a lowering of human APP/Aβ and human tau levels. Moreover, memory is improved and concomitant antidepressant-like effects are evident [41].

Among the drugs specifically predicted for AD, we found also spironolactone, with a high adjusted similarity and GSEA equal to 2 (Table 2). Spironolactone is an aldosterone receptor antagonist and mineralocorticoid receptor blocker approved for the treatment of hypertension and heart failure. It has been shown that this drug, in combination with eplerenone, attenuated Aβ -induced cognitive impairment [42]. The authors showed that the general improvement in cognitive function could be likely due to upregulation of BDNF levels in the frontal cortex and hippocampus, which may increase H2S, decrease Aβ, activate the Nrf2-dependent antioxidant system, and decrease neuroinflammation [42].

#### 3.2.3. GSEA Score 1

Among the drugs specifically predicted for AD, we found azacitidine, bezafibrate, diclofenac, and rifampicin with a high adjusted similarity and GSEA equal to 1 (Table 2).

Azacitidine belongs to the family of DNA methyltransferases inhibitors and was originally approved for myelodysplastic syndrome. Several studies have investigated the potential role of epigenetic modifications and in particular aberrations in DNA methylation as a biomarker in AD, and the role of DNA methyltransferases inhibitors as modulators of the methylation of AD risk genes [43,44].

Bezafibrate is a lipid-lowering fibrate, an agonist of PPAR-alpha, and is used to control hyperlipidaemia. It has been recently shown that it induces long-lasting positive effects on the sporadic AD model induced by STZ-ICV administration [45]. STZ-ICV leads to over-accumulation of Aβ and p-tau accompanied by neuroinflammation, decreased brain glucose utilization, neuronal loss, and cognitive impairment. This drug can cross the blood–brain barrier. As a result, it could improve cerebral glucose metabolism by activating PPARs and upregulating insulin sensitivity, as occurs in the peripheral system [45].

Diclofenac is a nonsteroidal anti-inflammatory drug that inhibits cyclooxygenase-1 and -2, and is used to treat the signs and symptoms of osteoarthritis and rheumatoid arthritis. This drug has been shown to both have active transport into the central nervous system and to lower amyloid beta. This is probably caused by a blockage or neutralization of IL-1β. From its comparison with two other nonsteroidal anti-inflammatory drugs, etodolac and naproxen (which has no effect on the development of AD), diclofenac has been found to be associated with a significantly lower frequency of AD [46].

Rifampicin is an antibiotic used to treat several types of mycobacterial infections via the inhibition of DNA-dependent RNA polymerase, leading to the suppression of RNA synthesis and cell death. It is able to reduce neurotoxic oligomers with a broad spectrum. This drug has been shown to inhibit the oligomer formation of Aβ and tau. The administration of the drug to aged APPOSK mice (Aβ oligomer model) leads to reduction in A β oligomers, as well as tau abnormal phosphorylation, synapse loss, and microglial activation [47]. Furthermore, there is also a reduction in the accumulation of the tau oligomer from the administration of rifampicin to aged tau609 mice (tauopathy model). Intranasal administration of rifampicin leads to higher drug levels in the brain than oral administration, for which it has already been shown to significantly reduce Amyloid β and tau pathologies in mice [48].

Among the drugs with GSEA score equal to 1, we found also three of those drugs belonging to the beta-blockers family (i.e., betaxolol, bisoprolol, and metoprolol) originally approved for hypertension treatment and already linked to a lower risk of developing AD [13], and diazoxide used to treat patients with hypoglycemia caused by excessive insulin levels. Diazoxide is a potassium channel activator capable of inhibiting the release of insulin from the pancreas. It can also reduce blood pressure (hypotensive) by arteriolar smooth muscle and vascular resistance. In a recent research study carried out on cell cultures of hippocampal neurons from mice, the authors showed that the combination of diazoxide and diphenyleneiodonium exhibited protective effects against amyloid β neurotoxicity [49]. Moreover, as an ATP-sensitive potassium channel opener, diazoxide has been shown to lead to a reduction in the accumulation of Aβ and hyperphosphorylated tau in the hippocampus and cerebral cortex in a mouse model of AD, with an improvement in cognitive functions [50].

Taken together, all these findings prompt us to believe that the network medicine approach to drug repositioning, as implemented by SAveRUNNER analysis, could significantly catalyze innovation in the discovery of promising repurposable drug candidates that deserve further investigation and experimental validation for AD.

## Figures and Tables

**Table 1:** Summary of disease–gene associations.

Disease	Number of Disease Genes	Database
Alzheimer’s disease	1749	Phenopedia
Amyloid neuropathies	8	Phenopedia
Behavior disorders	77	DisGeNET
Cardiomyopathy, dilated	148	Phenopedia
Choice behavior	33	Phenopedia
Executive dysfunction	33	DisGeNET
Huntington’s disease	79	DisGeNET
Language disorders	112	Phenopedia
Lewy Body disease	54	DisGeNET
Memory disorders	120	Phenopedia
Mild cognitive disorder	430	DisGeNET
Parkinson disease	629	DisGeNET
Pattern recognition, visual	31	Phenopedia
Supranuclear palsy progressive	43	Phenopedia

**Table 2:** Predicted drugs with their adjusted similarity value, GSEA score, and original medical indication from TTD [27].

Predicted Drug for AD	Adjusted Similarity	Drug Class	Original Medical Indication	GSEA Score	Shared with
regorafenib	0.99	multi-kinase inhibitor	Metastatic colorectal cancer	3	Cardiomyopathy, Dilated, Language Disorders
dexamethasone	0.99	corticosteroids	Rheumatoid arthritis	3	Memory Disorders
tamoxifen	0.98	estrogen receptor modulator	Breast cancer	3	Specifically predicted for AD
clopidogrel	0.99	platelet inhibitor	Thrombosis	2	Specifically predicted for AD
sirolimus (rapamycin)	0.99	mTOR inhibitor immunosuppressant	Organ transplant Rejection	2	Memory Disorders
everolimus	0.99	mTOR inhibitor	Kidney cancer	2	Specifically predicted for AD
gemfibrozil	0.98	PPAR-alpha agonist	Hyperlipidaemia	2	Specifically predicted for AD
spironolactone	0.95	aldosterone receptor antagonist	Congestive heart failure	2	Specifically predicted for AD
azacitidine	0.99	DNA methyltransferases inhibitor	Myelodysplastic syndrome	1	Specifically predicted for AD
bezafibrate	0.99	PPAR-alpha agonist	Hyperlipidaemia	1	Specifically predicted for AD
diclofenac	0.99	cyclooxygenase-1 and -2 inhibitor	Osteoarthritis	1	Specifically predicted for AD
rifampicin	0.99	DNA-dependent RNA polymerase inhibitor	Tuberculosis and Tuberculosis-related mycobacterial infections	1	Specifically predicted for AD
diazoxide	0.98	Potassium channel activator	Hyperthension	1	Memory disorders
betaxolol	0.85	Beta adrenergic antagonist	Hyperthension	1	Behavior disorders, cardiomyopathy, dilated, choice behavior, language disorders, memory disorders
bisoprolol	0.85	Beta adrenergic antagonist	Hyperthension	1	Behavior disorders, cardiomyopathy, dilated, choice behavior, language disorders, memory disorders, amyloid neuropathies
metoprolol	0.85	Beta adrenergic antagonist	Hyperthension	1	Behavior disorders, cardiomyopathy, dilated, choice behavior, language disorders, memory disorders, amyloid neuropathies

## Data Availability

All of the relevant data are within the manuscript and its Supporting Information files.

## Supplementary Materials

The following supporting information can be downloaded at: https://www.mdpi.com/article/10.3390/jpm12101731/s1, Table S1: Drug-disease network predicted by SAveRUNNER for all the analyzed diseases. Table S2: Candidate repurposable drugs predicted by SAveRUNNER for AD along with their original medical indication, the GSEA score, and the indication if that drug has been specifically predicted only for AD.
